# Universal Natural Shapes: From Unifying Shape Description to Simple Methods for Shape Analysis and Boundary Value Problems

**DOI:** 10.1371/journal.pone.0029324

**Published:** 2012-09-27

**Authors:** Johan Gielis, Diego Caratelli, Yohan Fougerolle, Paolo Emilio Ricci, Ilia Tavkelidze, Tom Gerats

**Affiliations:** 1 Department Biosciences Engineering, University of Antwerp, Antwerp, Belgium; 2 Radboud University Nijmegen, Section Plant Genetics, Institute for Wetland and Water Research, Faculty of Science, Nijmegen, The Netherlands; 3 Delft University of Technology, International Research Centre for Telecommunications and Radar, Delft, The Netherlands; 4 Université de Bourgogne, Laboratoire Le2i – UMC CNRS 5158, Le Creusot, France; 5 Campus Bio-Medico University, Rome, Italy; 6 I. Javakhishvili Tbilisi State University, I. Vekua Institute of Applied Mathematics, Tbilisi, Georgia; King Abdullah University of Science and Technology, Saudi Arabia

## Abstract

Gielis curves and surfaces can describe a wide range of natural shapes and they have been used in various studies in biology and physics as descriptive tool. This has stimulated the generalization of widely used computational methods. Here we show that proper normalization of the Levenberg-Marquardt algorithm allows for efficient and robust reconstruction of Gielis curves, including self-intersecting and asymmetric curves, without increasing the overall complexity of the algorithm. Then, we show how complex curves of k-type can be constructed and how solutions to the Dirichlet problem for the Laplace equation on these complex domains can be derived using a semi-Fourier method. In all three methods, descriptive and computational power and efficiency is obtained in a surprisingly simple way.

## Introduction

### Mathematics and the biological sciences

Understanding life is one of the major challenges for science in the 21^st^ century. Despite the exponentially growing mountains of data in the life sciences, in particular data from molecular biology, the challenge of developing geometrical models, always at the core in eras of scientific progress (Newton, Riemann, Einstein), remains completely open. Marcel Berger wrote explicitly [1]: *“Present models of geometry, even if quite numerous, are not able to answer various essential questions. For example: among all possible configurations of a living organism, describe its trajectory (life) in time”*. A free translation reads: we are nowhere near describing life mathematically, despite the numerous applications of mathematics in the life sciences.

In [2] the Russian mathematician I.M. Gelfand who had a great interest in biology, is quoted: “*There exists yet another phenomenon which is comparable in its inconceivability with the inconceivable effectiveness of mathematics in physics noted by Wigner - this is the equally inconceivable ineffectiveness of mathematics in biology.*” A geometrization of physics [3] seems to be a simpler task than a geometrization of biology.

A geometrization of biology, or more generally of nature, based on forms and formation of natural shapes (a geometrical theory of morphogenesis) is both an enormous challenge and a prerequisite for progress in science and the life sciences. René Thom wrote [4]: *“That we can construct an abstract, purely* geometrical theory of morphogenesis, *independent of the substrate of forms and the nature of the forces that create them, might seem difficult to believe, especially for the seasoned experimentalist used to working with living matter and always struggling with an elusive reality. This idea is not new and can be found almost explicitly in D'Arcy Thompson's classical book On Growth and Form”*.

Assuming that such geometrical theory exists and that there would be some analogy to past scientific theories in physics, as one could hope for, this geometrical theory will involve 1) *simple and uniform geometrical-mathematical descriptions*, coupled with 2) *natural curvature conditions*. Uniform geometrical-mathematical descriptions could involve once again conic sections, as before with Galilei-Kepler and Newton, or one-step transformations from the conics.

The importance of natural curvature conditions was pointed out by Schrödinger: *“The logical content of Newton's first two laws of motion was to state, that a body moves uniformly in a straight line ,…‥, and we agree upon calling force its acceleration multiplied by an individual constant. The great achievement was, to concentrate attention on the second derivatives – to suggest that they – not the first or third or fourth, not any other property of motion – ought to be accounted for by the environment”* [5].

### Commensurability, symmetry and Lamé-Gielis curves

Science has really focused on measurements and metric geometry with as fundamental question: “*how to measure, with what yardstick*?” While Euclidean and Riemannian geometry study geometry based on the Pythagorean theorem or quadratic forms, Riemann himself pointed out that other ways of measuring might be considered, e.g. fourth powers. Riemann's suggestion led to the development of Riemann-Finsler geometry. This will become a major topic in geometry in the 21^st^ century but was initiated in the early decades of the 20^th^ century by the successors of Riemann in Göttingen (Minkowski-Carathéodory-Finsler and Hilbert-Courant-Buseman), and developed further by various geometers [3] in particular by Shiing-Shin Chern [6].

Actually the fourth power is a particular example of so-called Minkowski metrics, which are distance metrics based on *Lamé curves* [7]. This family of curves includes superellipses (Eq. 1) and the conic sections (Equation 2–5). It is noted that power functions and power laws, ubiquitous in natural systems [8] simply are generalizations of parabola and hyperbola.
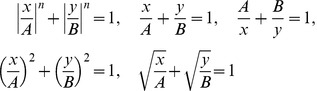
(1-5)


For a uniform description of natural shapes (the first step in a geometrization program) a geometrical approach with *Gielis curves*, *surfaces* and *transformations* has been proposed [9], [10], which generalize Lamé curves and surfaces for any symmetry. They provide for a single method of measuring a wide range of natural shapes, with measuring devices *adapted to the shape* [9], [10]. *Gielis transformations* (Eq. 6) operate on a function *f(ϑ)* and associated curves. For *f(ϑ)* constant we obtain transformations of a circle into square, starfish, hexagons, or self-intersecting polygons (for *m* = *p/q*).
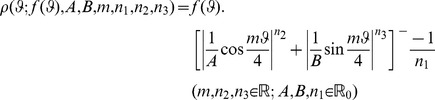
(6)


Since Lamé curves include all four conic sections [11], Gielis curves and transformations can also be considered as a one-step extension of conic sections. Gielis curves and surfaces (this name substitutes for the older name superformula [10], [12]) give a unique and uniform way of describing a wide variety of shapes as diverse as plant cells, stems and flowers, starfish, crystals, galaxies and the relativistic universe itself, hence the name *Universal Natural Shapes* [10]. Through Gielis transformations curves, surfaces and (sub-) manifolds all become commensurable or symmetrical as conic sections, in the spirit of Greek and modern geometry.

Indeed, the question of measuring and commensurability - the rationale behind both Lamé-Gielis curves and Riemann-Finsler geometry - is at the very heart of science and mathematics. Symmetry (-μετρια) for the Ancient Greek mathematicians means *proportion* or *right balance*, and συμμετρεω is the deliberate act of making objects *commensurable*, forming the real basis of mathematics and geometry. Going back to the basics of measuring and to the development of measuring devices and anisotropic geometries motivated from within geometry itself, we do not have to invent hypotheses.

### CAMC surfaces

Beyond a uniform description, the next challenge is to understand why shapes are the way they are. One answer is because they are the result of a variational problem. One example is Constant anisotropic mean curvature surfaces CAMC [12]. Constant mean curvature *H* (CMC, expressing uniform surface tension) surfaces are intimately connected to the Plateau problem and to conic sections [13]. In soap bubbles surface tension is distributed as uniformly as possible, serving as models or as equilibrium shapes for a wide variety of marine organisms [13].

**Figure 1:**
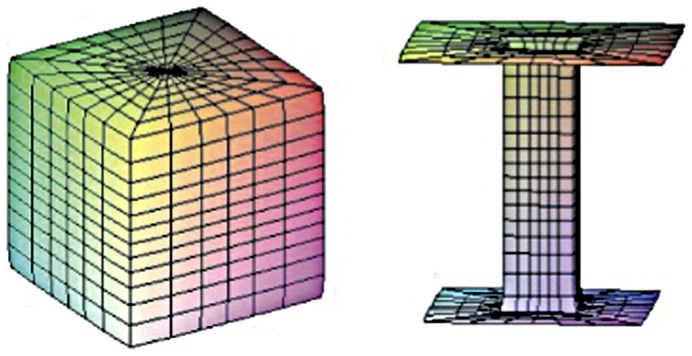
A supercatenoid (right) minimizing the anisotropic energy defined by a cube (left) among all surfaces having the same boundary.

CMC surfaces, however, are based on spherically isotropic energies. Constant anisotropic mean curvature (CAMC) surfaces, the anisotropic analogues of catenoids and Delaunay surfaces were studied using Lamé-Gielis surfaces as examples of Wulff shapes [12]. A Wulff shape is the “sphere” for an anisotropic energy in the sense that it is the minimizer of the energy for a fixed volume. The supercatenoid has the property that sufficiently small pieces of it minimize the anisotropic energy defined by the Wulff shape among all surfaces having the same boundary (Figure 1). Like catenoids in soap films minimize stress completely for isotropic energies, in supercatenoids stress is also minimized locally, defined by the anisotropic energy. Supercatenoids then provide equilibrium shapes for snowflakes and their development, taking into account the symmetry of ice. CAMC surfaces with Wulff shapes based on Lamé-Gielis curves open new ways of studying optimization in natural shapes.

### Goals of this paper

Chern's work [6] has made the general treatment of Riemann-Finsler geometry as easy as Riemannian geometry. Within this general framework the study of tangent spaces and curvatures based on Lamé-Gielis curves and surfaces in particular, could help elucidate the geometrical meaning of all curvatures in Riemann-Finsler geometry and the natural processes that are modelled in this way [10], (the second challenge of the geometrization program).

For the study of natural shapes however, in biology, physics and chemistry, there is also a more immediate need for practical computational methods to describe, analyze and compute shapes and their development. In this paper we show that Gielis curves allow for generalizing and simplifying existing methods rendering the practical study of natural shapes for scientists as easy as the general case of Riemann-Finsler geometry for geometers.

First, given certain natural shapes, biologists need ways to convert data points, obtained by measurements, into Lamé-Gielis curves. We present methods and algorithms to reconstruct Gielis curves and surfaces from data points and contours; the algorithm can also analyse self-intersecting shapes. Such shapes can be found for example in all projections in the plane of curves wound on helices or conics; in biology nucleic acids and proteins [14], [15] and phyllotaxis immediately come to mind.

Second, starting from single Gielis curves or surfaces, combined shapes can be constructed in a variety of ways. In this contribution we propose the method of a generalized Fourier series or partial sums of shapes and their associated trigonometric functions, which are no other than their own intrinsic coordinates.

Third, we present analytical solutions and computational results for a very classical boundary value problem of mathematical physics. Boundary value problems relate to the study of, among others, problems of heat distribution, vibrations in membranes and in elastic bodies. The background is that Gielis curves have opened the door to simplify computations of BVP of many types on any normal polar, spherical or cylindrical domain [16], [17]. Since almost all two and three-dimensional normal-polar domains are described (or at least approximated as closely as needed) by Gielis curves and surfaces, techniques have been developed with stretched polar coordinates for solving partial differential equations involving the Laplacian (including heat, wave, Laplace, Poisson, and Helmholtz equations) with boundary conditions of Dirichlet, Neumann or Robin type using a semi-Fourier method [16], [17]. Here we present the analytic Fourier-like solution to the Dirichlet problem for the Laplace equation on these combined domains.

## Materials and Methods

### Constructing potential fields for asymmetric Gielis curves with R-functions

Let m be defined as *m = p/q* with *p*,*q* natural numbers and relative prime. The parameter *p* represents the rotational symmetry number and the parameter *q* corresponds to the maximum number of self-intersections. For any point *P*(*x*, *y*), one can determine one intersection *I* between the curve and the half line [*OP*) as *I* = (*r*(*ϑ*) cos*ϑ*, *r*(*ϑ*) sin *ϑ*) with 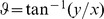, and we have *I* ^2^ = *r* ^2^. From this observation, an infinity of signed potential fields *F_i_*(*x*,*y*) such that *F_i_*(*x*,*y*) = 0 on the curve, *F_i_*(*x*,*y*)>0 inside the curve, and *F_i_*(*x*,*y*)<0 outside, can be defined as in [18], [19], [20], [21].

We present our results for the recovery of asymmetric rational Gielis curves (ARGC) constructed through multiple R-disjunctions of three potential fields presented in equations 7–9: *F*_1_ has been proposed by Gross *et al.* in [22] as radial distance for superquadrics, 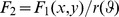 is the 2D equivalent of the function due to Fougerolle *et al.* in [19] for non self intersecting unit Gielis surfaces, and *F*_3_ has been suggested by Voisin in [21].
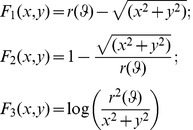
(7-9)


A technique to construct a potential field with desired differential properties for self intersecting Gielis curves, also known as rational Gielis curves (RGCs), has been proposed in [22] and relies on the combination of multiple potentials through R-functions [23]. The recovery of RGCs is a highly non-linear optimization problem in which we seek for the set of the parameters that minimizes the distances from a set of points to the curve. For our experiments, the symmetries are supposed to be known and we seek for the parameters *a*, *b*, *n*_1_, *n*_2_ and *n*_3_ in equation 6.

### Levenberg-Marquardt and a hybrid stochastic-deterministic algorithm

The most efficient methods in the literature apply the Levenberg-Marquardt's method, which is based on efficient approximations of the Hessian matrix and gradient of the cost/potential function. The key idea is to transform the potential fields such that they behave as an approximation of a distance function to the curve through normalization (in the sense of R-function normalization as introduced by Rvachev in [23]). Deeper insights on function normalization and R-functions can be found in [23] and [24].

A function f with non-vanishing gradient can be normalized to the first order as:
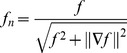
(10)The *i-th* component of the gradient of *f_n_* can be written as:
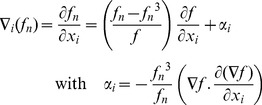
(11)A convenient approximation can be obtained by setting α*_i_* = 0. In such a case, 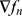 is approximated by 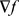 scaled by a factor (*f_n_*−*f_n_* ^3^)/*f*.

A Levenberg-Marquardt iteration requires to solve
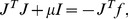
(12)where μ is a regularization coefficient which is increased when the iteration fails and decreased otherwise, and 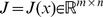 the Jacobian of 
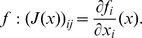
 In the normalized case, the system becomes
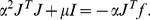
(13)Let λ_1_ and λ_2_ be the smallest and largest eigenvalues of *J^T^ J*, respectively. The matrix A = *(J^T^ J+μI)* is positive definite symmetric and
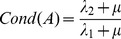
(14)Similarly, the conditioning of matrix *A_α_ = (α^2^ J^T^ J+μI)* is
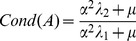
(15)It is important to observe that 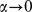 when 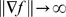, and that α is always well defined because 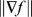 and *f* cannot be null simultaneously, by definition. As a consequence, while matrix *A* might be badly conditioned due to large gradient magnitude, the matrix *A_α_* has a conditioning which tends to *1*, thus the normalized algorithm produces more trustful estimates in presence of strong gradient magnitudes.

A hybrid algorithm uses a fast evolutionary algorithm for initialization with the shortest Euclidean distance replacing the potential fields proposed in literature for a better discrimination between individuals. Once an initial guess, *i.e.* with coherent symmetries and pose, is obtained, the normalized Levenberg algorithm efficiently determines the optimal shape parameters.

### (Partial) Fourier-like sums of Lamé-Gielis curves

The coordinate functions of supercircles and superellipses are obtained when *f(ϑ)* is cosine or sine, and sums can then be constructed. It can be shown that these are the coordinates functions of Gielis curves. We can then construct sums of the shapes and their coordinates functions, whereby these coordinate functions can be inscribed in anisotropic spaces.

More generally a Fourier-like series (Equation 16) can be constructed, in which every term of the series is inscribed in an anisotropic space. The summation can be infinite or finite. In the latter case we speak of partial sums. We refer to k-type Lamé-Gielis curves for a partial sum with k terms, with k a natural number.
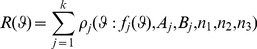
(16)


We emphasize that all two-dimensional normal-polar domains may be described or approximated accurately by selecting suitable modulator functions and parameters.

### The Dirichlet problem for the Laplace equation

To define the Laplacian in stretched polar coordinates, we introduce the stretched radius 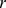 such that:
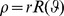
and the following curvilinear co-ordinates 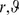 in the *x,y* plane with 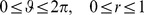,
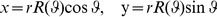
(17)The Laplace operator is defined in the new stretched co-ordinate system *r*, *ϑ* as:
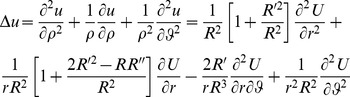
(18)For 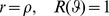 we recover the Laplacian in usual polar co-ordinates.
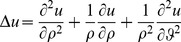
(19)


The interior Dirichlet problem for the Laplace equation in a starlike domain *D*, whose boundary is described by the polar equation 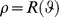 is:
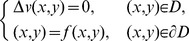
(20)In a similar way, the exterior Dirichlet problem subject to the null condition at infinity 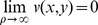 may be addressed:
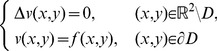
(21)


## Results

### Analysis of self-intersecting shapes

Numerous problems deal with least-square minimization of non linear models with n parameters from m observations (m>n). For the recovery of Gielis curves, when using the *l_2_*-norm, one seeks for a local (or global) minimum of a function *F(x)* defined as:
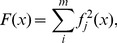
(22)The function *f_j_(P)* is a potential field such that *f_j_(P) = 0* if the point *P* lies on the curve, as defined in equations 7–9. The key idea of normalization is to transform the highly non linear potential fields *f_j_(P)* with non vanishing gradient at their zero set, into approximations of distance, using
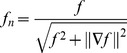
(23)and its core elements are the evaluation of the function *f_n_*, *i.e.* the evaluation of *f* and its gradient 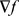. More precisely, the gradient of the normalized function can be written as
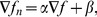
(24)

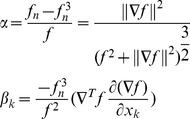
(25)


Thus, for an exact computation of *f_n_* the second order partial derivatives must be computed. A convenient approximation can be obtained by setting β to zero, which corresponds to an approximation to the first order. In such a case, *f_n_* is approximated by the gradient of the original function scaled by a factor α. The justification of this approximation is two-fold:

1. The gradient is the core element of the Levenberg-Marquardt algorithm. In its implementation, the second order partial derivatives are also removed for stability reasons and efficiency, as it is well admitted by the community. As an illustration, the Hessian matrix is commonly approximated as *J^T^ J*.
2. By definition, the function *f* and its normalized version *f_n_* share a common iso value: their zero set, *i.e.* the locus in space where both functions are null, is the same. Therefore, on the boundary of the domain, both gradients are collinear, orthogonal to the curve and thus only differ by the scale. Consequently, the considered approximation is valid and accurate on the boundary of the domain.

**Figure 2:**
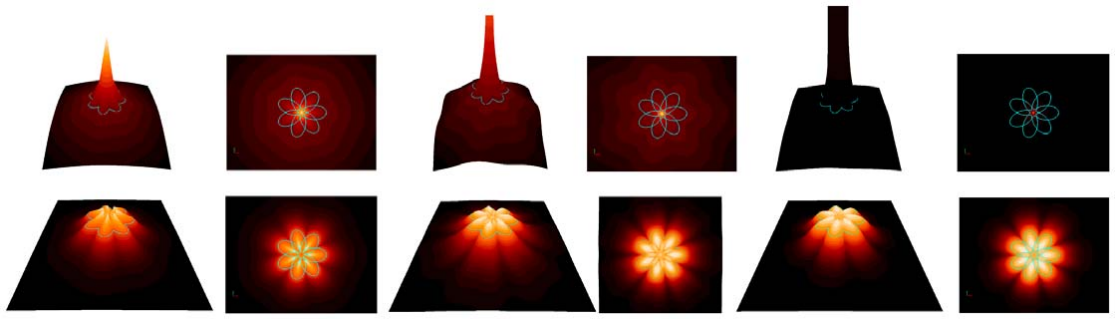
Relative intensity of the functions *F*_1_, *F*_2_, and *F*_3_ generated with A = *B* = 1, *n*_1_ = 0.25, *n*_2_ = *n*_3_ = 3.5, *p* = 7, and *q* = 3. The RGC is represented in light blue. Line 1: Original functions. Line 2: Normalized functions. From left to right *F*_1_, *F*_1_ top view, *F*_2_, *F*_2_ top view, *F*_3_, and *F*_3_ top view.

Figure 2 illustrates the intensity of the three considered potential fields as well as their normalized version.

**Figure 3:**
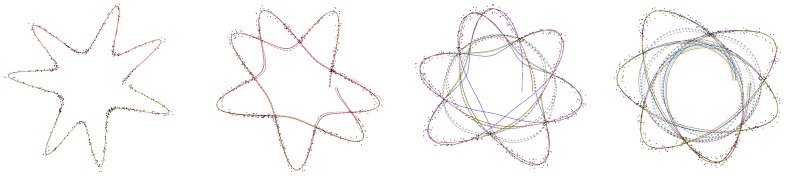
Reconstruction results using standard LM and normalized LM. Reconstructed curves from noisy synthetic data for A = 0.8, B = 1.2, *n*_1_ = 2.5, *n*_2_ = 5, *n*_3_ = 15, *p* = 7, and *q* = 1,…, 4. In dashed line: original functions, in solid line: normalized function.

As illustrated in figure 3, the standard Levenberg-Marquardt falls into local minima once the number of self-intersection increases, whereas the normalized algorithm remains able to correctly reconstruct curves with higher self-intersection. The reconstruction results of the standard and normalized Levenberg approaches are drawn in dashed lines and solid lines, respectively. Since the potential field of a self intersecting curves is built from multiple R-functions of elementary potential fields, *i.e.* is a non linear combination of multiple potential fields, this illustrates the ability of the algorithm to reduce the effect of severe non-linearities of the problem.

We have considered the worst case for our experiments, *i.e.*, non-symmetric scales, non-symmetric shape coefficients, noisy synthetic data, and we observe the quality of the reconstructed curves with the increase of the number of self-intersections *q*. In figure 3(a), *q* = 1 so the curve is not self-intersecting, which corresponds to the simplest case. The curve is accurately reconstructed for the six functions. In figure 3(b), *q* = 2: we now minimize the R-disjunction of the RGC with itself, and the six functions still lead to equivalent results with very slight differences.

In figure 3(c), *q* = 3, which corresponds to the double R-disjunction of the RGC with itself. For two self-intersections the standard algorithm converges to local minima whereas the normalization allows for correct reconstruction. Figure 3(d) presents similar results for *q* = 4. For three self-intersections the standard algorithm converges to local minima whereas the normalization allows for correct reconstruction. For *q*>4, all approaches systematically lead to convergence to local minima.

### The influence of noise and presence of outliers

In this section several reconstruction results illustrate the efficiency and the robustness of our hybrid algorithm. As a convention, the reference curve, which has been used to generate the data is drawn in red and the recovered curve is in blue. The following tables illustrate the global behaviour of the three approaches and have been obtained through the analysis of several thousands of random data sets. The evolutionary algorithm uses the shortest Euclidean distance to compute the cost function in equation (22). The LM algorithm uses the potential fields defined in equations 7, 8 and 9, and as a remark, all three potential fields lead to equivalent reconstruction results.

Table S1 illustrates the behaviour of the algorithm when data are degraded. The results illustrated for the Normalized LM algorithm have been obtained by manually setting the exact symmetries. The combination of an initialization using EA followed by LM systematically outperforms both approaches used separately. Each row of the Table S1 illustrates some fundamental characteristics of the method:

- If the data are fully spread all around the center of gravity, all three methods lead to accurate results. In such case, it is therefore most appropriate to use the LM algorithm to reconstruct the curve.
- Even a slight variation of the initial pose might lea
- d the LM algorithm to converge to a local minimum, whereas the EA and EA+LM algorithm still correctly reconstruct the data.
- Rows 3 and 4: the evolutionary algorithm cannot accurately capture the details of the curve, and sometimes leads to incorrect symmetry detection. Here, the reference curve used to generate the data has 6 (row 3), and 8 (row 4) rotational symmetries, and the EA algorithm, after only 300 iterations, ends with a local minimum obtained with only 2 and 4 symmetries at row 3 and 4, respectively. Despite of this inappropriate initialization of the symmetries, the combination EA+LM still improves the accuracy and leads to an acceptable reconstruction, whereas the LM only converges to a local minimum.
- If data are strongly degraded, then several degenerate curves (close to arc circles) can approximate the data. In such case, all three approaches lead to equivalent results.

Table S2 illustrates the fact that the recovered curves tend to the average circle when data contain outliers. This phenomenon is naturally amplified when data are degraded as presented in Table S1. The influence of the noise is less critical then outlier presence, since the errors are spread uniformly. As a matter of fact, all three algorithms are able to accurately reconstruct the data, the only difference being the optimal value of the cost function in equation (22) to be larger with higher noise intensities.

Table S3 presents executions time for the evolutionary, Levenberg-Marquardt algorithms and their combination. The evolutionary algorithm has a linear complexity in function of the population size and the number of iterations, so the results presented correspond to the computational time for one iteration divided by the population size. The last column corresponds to the total execution of the Levenberg-Marquardt algorithm. Using SED leads to more accurate and robust results but approximately multiplies the computational cost by a factor 5. For our experiments, we have used a population of 30 individuals and a maximum number of 300 iterations, which leads to a maximum total execution of 3 minutes at the worst cases. By comparison, the deterministic method is more efficient since it only represents few seconds in total.

### Gielis curves of k-type

In a generalized Fourier-like series (eq. 16), i.e., on any term of a classical Fourier series a Gielis transformation can act, any Lamé-Gielis curve is encoded directly, in one term only. This is of course a direct consequence of the fact that they are encoded in one equation, and differ from the circle only in a few parameters. In a similar way this can be extended to spherical harmonics, since surfaces (such as starfish, pyramids, cones and flowers or highly complex shapes) differ from a sphere only in a few parameters.

This can further be used as a starting point for building curves and surfaces as sums. Partial sums are then called of finite k-type with k integer. Some special curves like Rhodonea (flower) curves, cardioids and limacons are of k-type with k = 2. Cardioids have been shown as a good model for *Arabidopsis* leaves [25] and superformular modifications of the cardioid were used to describe leaves of *Hydrochoris morsus-ranae*, *Fagopyrum tataricum*, *Polygonum convolvulus*, *Rumex acetosella* and *Hedera saggitifolia* [26].

**Figure 4:**
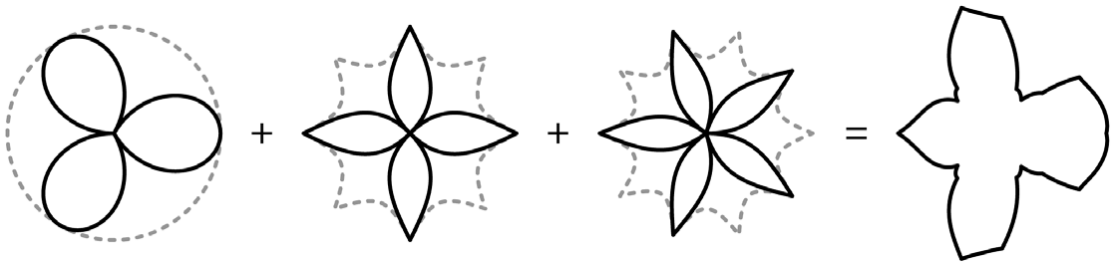
k-type Gielis curve with k = 3 for 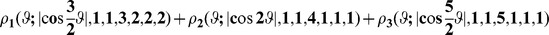
 ****.

Here we present one example of 3-T with three terms. The first term is a three-lobed flower in an isotropic space, since exponents *n_i_* = 2 yields a Euclidean circle. The second and third term are a four and five lobed flower inscribed in a square and pentagon, respectively, with exponents *n* = 1. In the shape outline of a flying bird one can indeed observe the various symmetries (Figure 4).

### The Laplace equation for k-type curves

For studying the Dirichlet problem for the Laplace equation using k-type curves, the following theorem is proved:

*Theorem* 1- *Let* 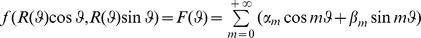,

where
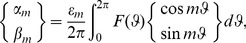
(26)

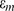
 being the usual Neumann's symbol. Then, the interior boundary-value problem for the Laplace equation admits a classical solution
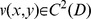
such that the following Fourier like series expansion holds:
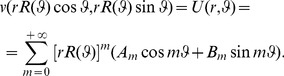
(27)The coefficients 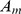, 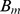 can be determined by solving the infinite linear system
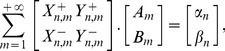
(28a)where
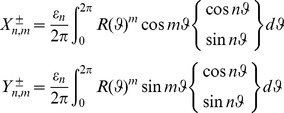
(28b)with 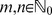.

**Proof:**

In the stretched co-ordinates system, for the 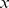, 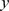 plane the dominant 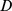 is transformed into the unit circle; so, the usual eigenfunction method and separation of variables with respect to the variables 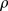 and 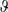 can be used. As a consequence, elementary solutions of the problem can be searched in the form
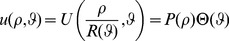
(29)Substituting into the Laplace equation we easily find that the functions 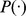, 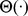 must satisfy the ordinary differential equations
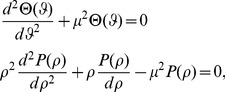
(30)respectively.

The parameter 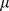 is a separation constant whose choice is governed by the physical requirement that any fixed point in the plane the scalar field 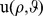 must be single-valued. So, by setting 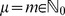 we find
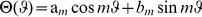
(31)where 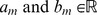 denote arbitrary constants.

The radial function 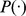 can be readily expressed as follows:
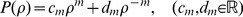
(32)As usual we assume 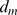 for the boundedness of the solution. Therefore, the general solution of the interior Dirichlet problem can be searched in the form
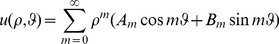
(33)Finally, imposing the boundary condition
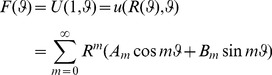
(34)and using the Fourier projection method, the solutions follow.

REMARK 1- Let us consider the associated interior Dirichlet problem for the Laplace equation on the unit circle with boundary values 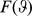. The solution of such a problem is readily expressed as:
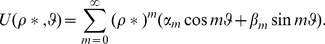
(35)By virtue of the maximum principle, the assumption 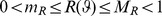 implies that the solution of the problem (20) is dominated by (35). Therefore,
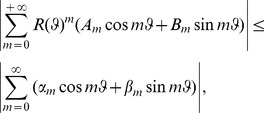
(36)and using the linearity of the operator, we find
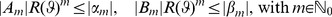
(37)By Lebesgue's theorem, the Fourier coefficients 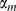, 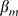 must go to zero when 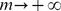 and the order of convergence to zero increases with the smoothness of boundary values 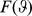. According to inequalities (37) the coefficients 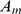, 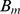 are also infinitesimal, since 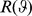 is bounded. This means that the vectorial operator defined by the system (28) is compact. In fact we can split up this operator in the sum of two parts, such that the former is finite-dimensional and the latter features maximum (or 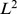) norm as small as we wish.

In the same way the exterior Dirichlet problem
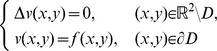
subject to the null condition at infinity
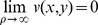
may be addressed. In particular the following theorem can easily be provided:

*Theorem* 2- Under the hypotheses of theorem 1, the exterior boundary value problem for the Laplace equation admits a classical solution
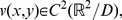
such that the following Fourier like series expansion holds:
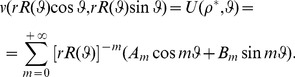
(38)The coefficients 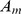, 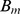 are the solution of the infinite linear system
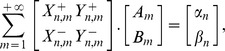
where
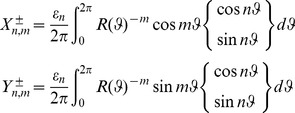
(39)with 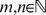.

REMARK 2: The formulas still hold true under the assumption that the function *R(ϑ)* is a piecewise continuous function and the boundary data are described by square integrable functions, not necessarily continuous, so the relevant Fourier coefficients *α_m_*, *β_m_* are finite quantities.

**Figure 5:**
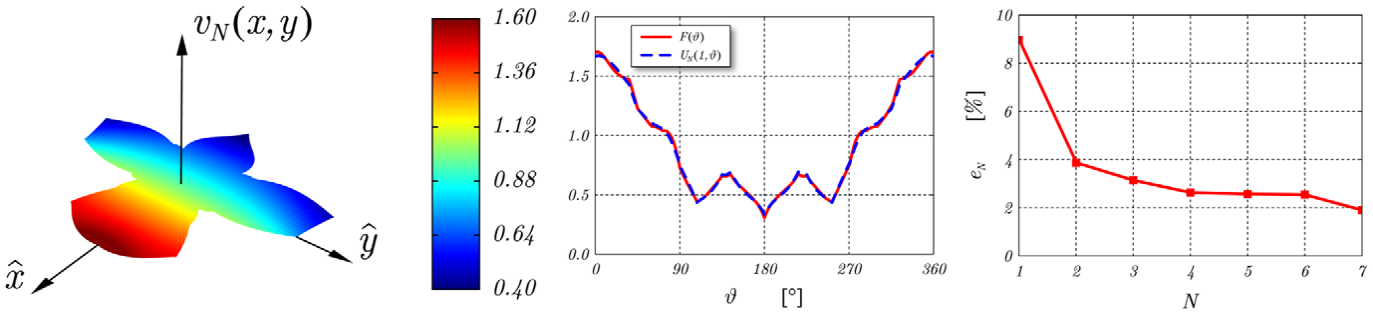
Left: Spatial distribution of the partial sum *U_N_* of order N = 7 representing the solution of the Dirichlet problem for the Laplace equation for the domain described in **Figure 3** and with *f(x,y)* = *x*+*cos y* describing the boundary data. Center: Angular behaviour of the partial sum *U_N_(1,φ)* with expansion order N = 7. Right: Relative boundary error *e_N_* for N = 7.

To assess the performance of the technique in terms of accuracy and convergence rate, the relative boundary error is evaluated using Equation 40 with 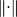 denoting the usual *L^2^* norm, *U_N_* the partial sum of order N relevant to the Fourier-like series expansion representing the solutions of the Dirichlet problem for the Laplace equation, and *f(x,y)* the function describing the boundary values (Figure 5).
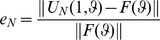
(40)


## Discussion and Conclusion

### Analysis

The approaches proposed in the literature for Gielis curve and surface recovery can be classified in two families: the deterministic approaches proposed by Fougerolle *et al.* [18], [19], [23], and stochastic approaches proposed by Bokhabrine *et al.* [20], and Voisin [21]. Both existing techniques do not handle asymmetric curves (only unit supershapes are considered in [20]) nor self-intersecting curves or surfaces, because no implicit field for such objects existed at that time. Deterministic approaches are highly sensitive to initialization (pre-segmented data and empirical initialization of pose and symmetries) and might often converge to local minima in presence of noise or incomplete data, whereas the stochastic approaches are more robust, but are time consuming due to the lack of discrimination between individuals implied by the non-linear potential fields proposed in [19], [20]. More recently, a technique to build implicit fields with guaranteed differential properties for asymmetric self-intersecting Gielis curves and surfaces has been proposed in [23].

In this paper we have presented a robust and efficient modification of Levenberg-Marquardt's algorithm for the recovery of asymmetric rational Gielis curves. This modification is efficient since the inner structure of Levenberg-Marquardt remains unchanged and only scale factors are introduced. This technique improves the robustness of the method, leads to convergence to optimal result with higher symmetries, and allows for better comparison between different functions since it rescales them to the same interval ]−1,1[ while also guaranteeing a similar behaviour near the zero set of the cost function. The minimization of the normalized function only requires one extra computation of the scale factor 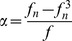 for each data point, thus does not increase the time or complexity of the algorithm.

Moreover, the hybrid algorithms encompasses and improves all the existing approaches: we propose a fast evolutionary algorithm for initialization in which the shortest Euclidean distance replaces the potential fields proposed in literature for a better discrimination between individuals. But instead of running the process over a (very) long time to reach a nearly optimal solution, the algorithm only performs a reduced number of iterations, because it appears that during the very first iterations of the algorithm, the individuals with non appropriate symmetries and incoherent poses and scales are efficiently discarded, which avoids the initialization issues. Once an initial guess, i.e. with coherent symmetries and pose, is obtained, the normalized Levenberg algorithm efficiently determines the optimal shape parameters. As a consequence, the proposed algorithm benefits from the robustness to initialization of stochastic approaches and still remains efficient since the fine tuning of the shape and scale parameters is handled by an extension of the deterministic approaches which is able to reconstruct self intersecting asymmetric curves or surfaces.

The ability to determine a Gielis curve representing complex data opens new perspectives in various research areas such as engineering, computer vision, crystallography, biology and physics, etc. In recent publications Lamé and Gielis curves and surfaces have been used, among others, in medical imaging [26], [27], [28], to study the cells in dielectric properties of cells in suspension [29], mechanical strength of leaf petioles [30], antenna technology [31], [32] and nanotechnology [33]. The robustness of methods, even under high noise levels and for self-intersecting curves, can have significant advantages, whenever measurements are involved and interpolations of data points.

In biology self-intersecting curves are obtained in all cases where helical or spiral structures are projected onto a plane. A variety of polygons and star polygons with integer and non-integer symmetries, respectively are found in nucleic acids, proteins, viruses and quasicrystals as shown by A. Janner in [14], [34]. [35]. In a whorled configuration sepals of rose for example still display a spiral background, resulting in self-intersecting shapes with m = 5/2.

The remaining challenges include the extension of the algorithm to surfaces, and the extension of dimensionality of the research space to handle translation, scaling, rotation and global deformations.

### Gielis curves of finite k-type

Gielis curves and surfaces allow for a uniform description of natural shapes in an extremely compact way. From the point of view of information theory the complexity of a collection of LG curves and surfaces, is drastically reduced. Resulting from a single equation circles, squares, starfish and a wide range of natural and abstract shapes differ in a few variables only. Describing a wide variety of shapes has hitherto been an intractable problem without resorting to infinite series. One very famous series for describing shapes is the Fourier series for periodic waves and elliptic Fourier descriptors for closed biological shapes in the field of morphometrics [36]. With Gielis curves and their coordinates in equation 10 we have a generalized Fourier series that allows us to express shapes in extremely compact ways.

The notion of k-types is based on Chen's finite type curves, which are of infinite or of finite type, depending on whether their Fourier expansion is infinite or finite [37]. From this geometrical perspective, there is one and only one closed curve that can be expressed in a finite Fourier series with respect to arc length, and that is the circle itself.

This theorem implies that the circle is the only closed planar curve that is of finite type, namely of 1-type (*1T*) with all cosine and sine terms equal to zero, but any other curve necessarily has a Fourier expansion of infinite type (*∞T*). An alternative interpretation is that all curves other than the circle, including the ellipses, are equally *complex*: once their Fourier expansion starts, it never stops [38]. Their expansion contains infinitely many terms (*∞T*).

Instead of infinite series, however, truncated for practical reasons, direct description of shape with Gielis curves provides a finite approach. Obviously, when Gielis curves are used as unit circles in a generalized Fourier series, i.e., on any term of a classical Fourier series a Gielis transformation can act, any Gielis curve is encoded directly, in one term only. They are of one-type (*1T*) and their expansion, once it starts, stops immediately. Hence, all Gielis curves, including the circle and Lamé curves, are equally *simple*.

Beyond these simple curves Gielis curves of k-type (with k sufficiently small) can be constructed as a possible way of combining different shapes, leading to a canonical way of spectral decomposition of shapes in terms of their own coordinate systems. This can have various consequences in science and technology, not only conceptually, but also computationally. A wide range of natural shapes can simply be encoded in a few numbers, based on intrinsic coordinate systems, adapted to the shape. A major challenge is to develop analytic tools that allow the direct use of a generalised Fourier series in image and curve recognition. It is noted that the classical series and transforms that have been used widely and for almost two centuries, are reduced to special cases.

Gielis k-type curves might unveil basic symmetries in higher organisms, symmetries that would remain hidden otherwise. Symmetries of 3, 4 and 5 are observed in the overall shape of a flying bird (Figure 4). These same basic symmetries (3, 4 and 5) are observed widely in natural shapes, for example in marine diatoms [39], [40] and in square bacteria thriving in highly saline environments [41]. In the evolution of angiosperms with a tendency to evolve from polymery (associated with spiral phyllotaxis) to oligomery (associated with whorls), trimery is the rule in the monocots while in eudicots it is pentamery [42].

In higher animals these basic symmetries might be combined in some way, for example with k-type curves as in Figure 4. While speculative at this stage, it may provide a direction for further investigations into the Bauplan of birds, reptiles and mammals, in which growth from a central point within enclosing forms may help to understand shape, development and developmental/evolutionary stability. We note that within the framework of Gielis curves associated invariances can be studied; for given shape parameters (exponents in Eq. 6) area remains invariant when symmetry is changed from m = 4 to m = 3 (yielding convex triangular shapes) or from m = 4 to m = 5 (concave pentagons) [9]. The latter case can be observed in Figure 4 where the enclosing forms of the second and third term (with exponents all equal to 1) have the same area.

### Laplace equation on k-type Gielis domains

Techniques were developed with stretched polar coordinates for solving partial differential equations involving the Laplacian (including heat, wave, Laplace -, Poisson -, and Helmholtz equations) with boundary conditions of Dirichlet, Neumann or Robin type using a Fourier method for Gielis domains [43], [44]. As an extension, we obtain the analytical solution of the interior and exterior Dirichlet problems for the Laplace equation in Gielis domains of k-type computed here for the shape of Figure 4.

Highly accurate approximations of the solution, featuring properties similar to the classical ones, are obtained. The *L^2^* norm of the difference between the exact solution and its approximate values is generally small. The point-wise convergence property of the solution seems to be in good agreement with the theoretical findings on series expansions by Lennart Carleson [45], with only exception of a set of measure zero formed by cusped and quasi-cusped singularities of the boundary.

One general and coherent method, giving closed form solutions for any such domains, thus substitutes for a variety of methods (such as Green's functions approximation by least squares techniques, conformal mapping or solution of the boundary integral equation by iterative methods) avoiding the cumbersome computational methods of finite differences and finite elements. Closed form solutions of a wide range of classical differential problems, in planes and solids are possible, also for multi-valued functions as in Riemann surfaces [16] or self-intersecting (rational) Gielis curves. The method can readily be extended to shapes described with Fourier descriptors, a method widely used to describe very complex shapes in biology.

The simple computational method for obtaining solutions of BVP using Fourier methods combines the ideas and insights of Gabriel Lamé (1795–1870) and Joseph Fourier (1768–1830), both professors at the Ecole Polytechnique in Paris. In 1817 Gabriel Lamé published his remarkable book [6], proposing superellipses (Eq. 2 with A = B and n = 1) as a model for crystallography. In his later works in mathematical physics Lamé envisaged that, from a mathematical point of view, to study a physical system amounts to the study of curvilinear coordinates, representing the given physical situation. Hence, the mathematical world of curvilinear coordinates may be regarded as a model of the world of physical systems [46]. To study the physical problem (Lamé for example worked on heat distribution and elasticity problems) adapted with a suitable system of curvilinear coordinates, only one equation needs to be solved: the Poisson equation in curvilinear coordinates, with appropriate boundary conditions; other equations and laws are reduced to special cases [46].

This solution can now be obtained using Fourier methods and generalized to a variety of BVP [43], [44]. Moreover, Gielis curves and surfaces carry natural curvilinear coordinate systems adapted to the system under study. The study of for example strength, heat distribution or vibration analysis, can all be computed in this way. Fields of applications include, amongst others, solid-state physics, fluid dynamics, electromagnetism, telecommunications, quantum theory, signal analysis, chemistry, economics and finance, plants and flowers, lower animals and, perhaps, the study of the Bauplan of animals. In a more general way, it allows the study of manifolds with boundaries with corners or conical singularities, bridging the discrete and the continuous.

We note a direct connection between k-type curves, Laplacians, boundary value problems and CAMC. For the study of natural shapes considered as physical submanifolds, from a geometrical point of view, the Laplacian is directly related to the mean curvature *H*, which is a measure for the surface tension a shape receives from a surrounding space. Interestingly, in Chen's k-type theory the Fourier expansion of a curve with respect to arc length is nothing but the spectral decomposition of the curve with respect to its Laplacian [37], [38]. It remains to be studied how k-type Gielis curves correspond to stationary solutions of certain functionals in the same way as k-type curves and surfaces were studied in the framework of optimal submanifolds. CAMC surfaces are a first step in this direction, providing for a class of shapes that can be used as equilibrium shapes in non-equilibrium conditions, for example in the formation of snowflakes and the development of flowers.

### Universal Natural Shapes and Science Rationelle Unique

Gielis transformations, which are essentially a generalization of the Pythagorean Theorem and of conic sections, allow for a uniform description of a wide range of abstract and natural shapes, opening the door for a geometrical theory of morphogenesis, which is similar to a geometrization of nature (not only of physics). In a geometric way Gielis curves make natural shapes, objects and phenomena commensurable (i.e. symmetric).

The discovery of Gielis transformations as a method of measuring for natural shapes amounts to the introduction of coordinates adapted to the shapes. These coordinates induce certain metrics on the surface or boundary and in the tangent spaces. Using tangents, tangent spaces and curvatures based on Gielis curves could unveil the geometrical meaning of all curvatures in Minkowski and Riemann-Finsler geometry and the various process that are modelled in this way [3], [8], [9], [47], [48], [49], [50], [51], [52], [53]. Lamé's Science Rationelle Unique (which is mathematical physics), Universal Natural Shapes and a geometrical theory of morphogenesis all resonate along the same lines of providing a geometrical picture of the world. In a general geometric framework this will concern also the theories of ideal submanifolds, including finite type surfaces [37] and of constant ratio submanifolds [52].

In this paper we have shown that dedicated computational techniques can be developed based on classical approaches, Levenberg-Marquardt for analysing data and point clouds, and Fourier techniques for obtaining accurate solutions for boundary value problems. The Levenberg-Marquardt algorithm can be made faster, more efficient and more robust by the proposed normalisation. Concerning Fourier analysis we note that it is only since 1966 that the theoretical foundations of Fourier analysis have been secured [45], and that it is only since 2007 that they can be applied in a very general way on any normal domain (including composite domains) in two and three dimensions. Very good results are obtained already for low expansion order N. In the same spirit k-type Gielis curves with k finite render all considered shapes (curves and surfaces) equally simple. These methods will be of great value in studying the way natural shapes develop and grow.

## Supporting Information

Table S1 Quality of the recovered curves with incomplete data.
(ZIP)

Table S2 Robustness to outliers.
(ZIP)

Table S3 Computational times for evolutionary and deterministic algorithms, in seconds.
(ZIP)
