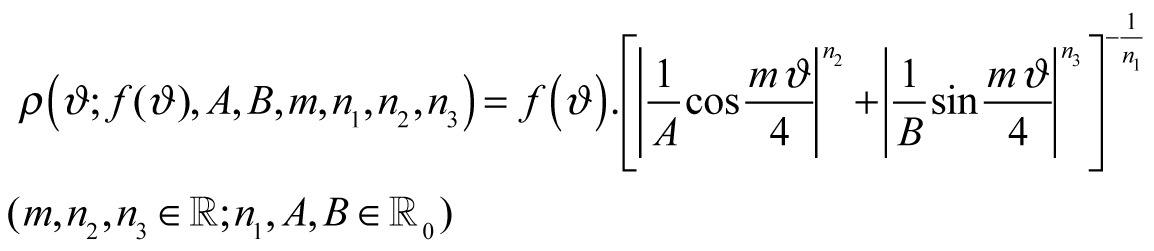# Correction: Universal Natural Shapes: From Unifying Shape Description to Simple Methods for Shape Analysis and Boundary Value Problems

**DOI:** 10.1371/annotation/2a59cb22-644c-40fd-b4eb-6367e3974968

**Published:** 2012-11-09

**Authors:** Johan Gielis, Diego Caratelli, Yohan Fougerolle, Paolo Emilio Ricci, Ilia Tavkelidze, Tom Gerats

In the Introduction, in the section titled "Commensurability, symmetry and Lamé-Gielis curves", there was an error in Equation 6. Please view the complete, correct equation here: