# Supplementary figures and images for: Universal Natural Shapes: From Unifying Shape Description to Simple Methods for Shape Analysis and Boundary Value Problems
Source: PLoS One. 2012 Sep 27;7(9):e29324. doi: 10.1371/journal.pone.0029324 (PMC3459917; doi:10.1371/journal.pone.0029324)

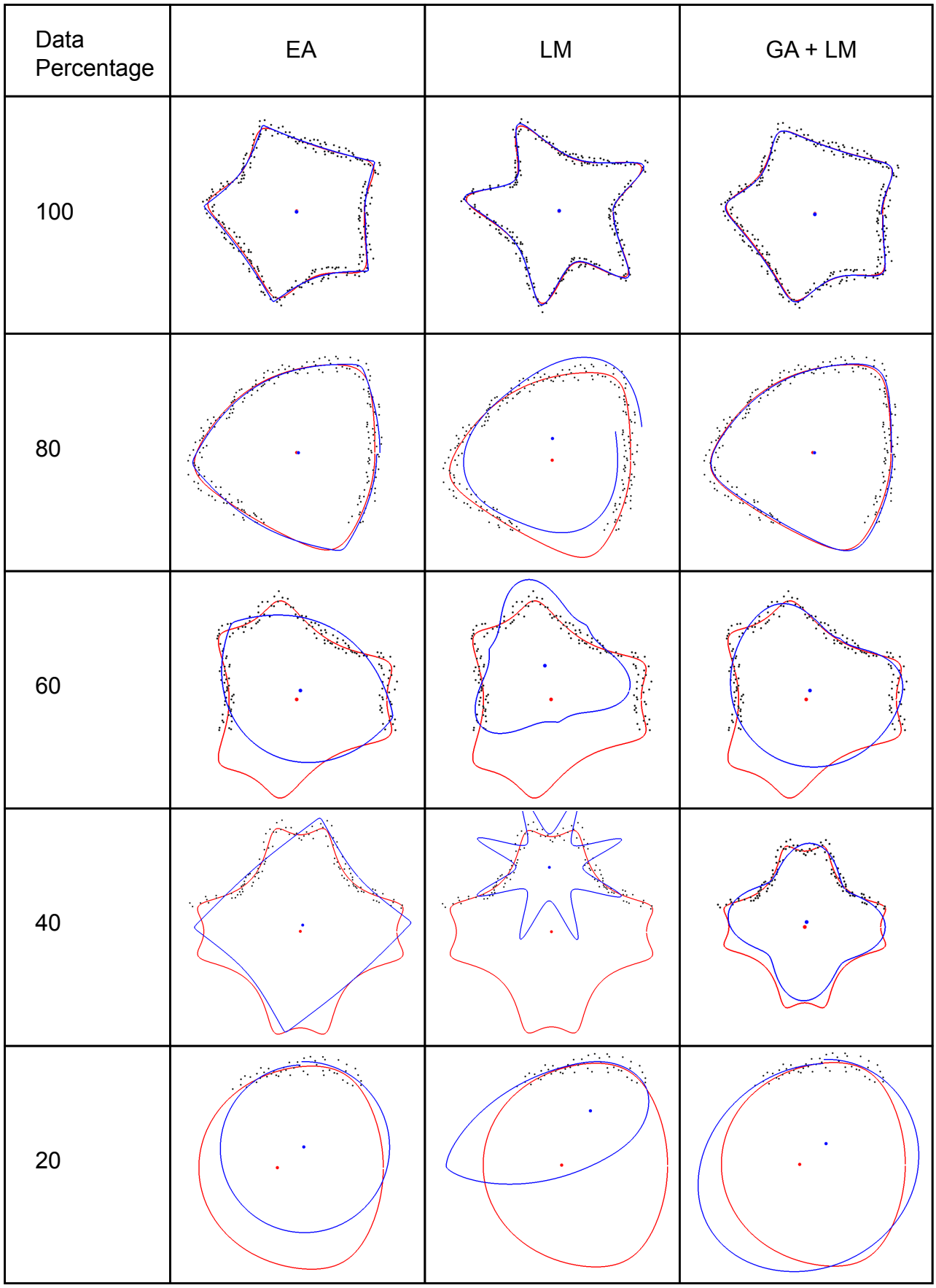

Supplement: Table S1 — Quality of the recovered curves with incomplete data. (ZIP) [file pone.0029324.s001.zip › Table S1.pdf]

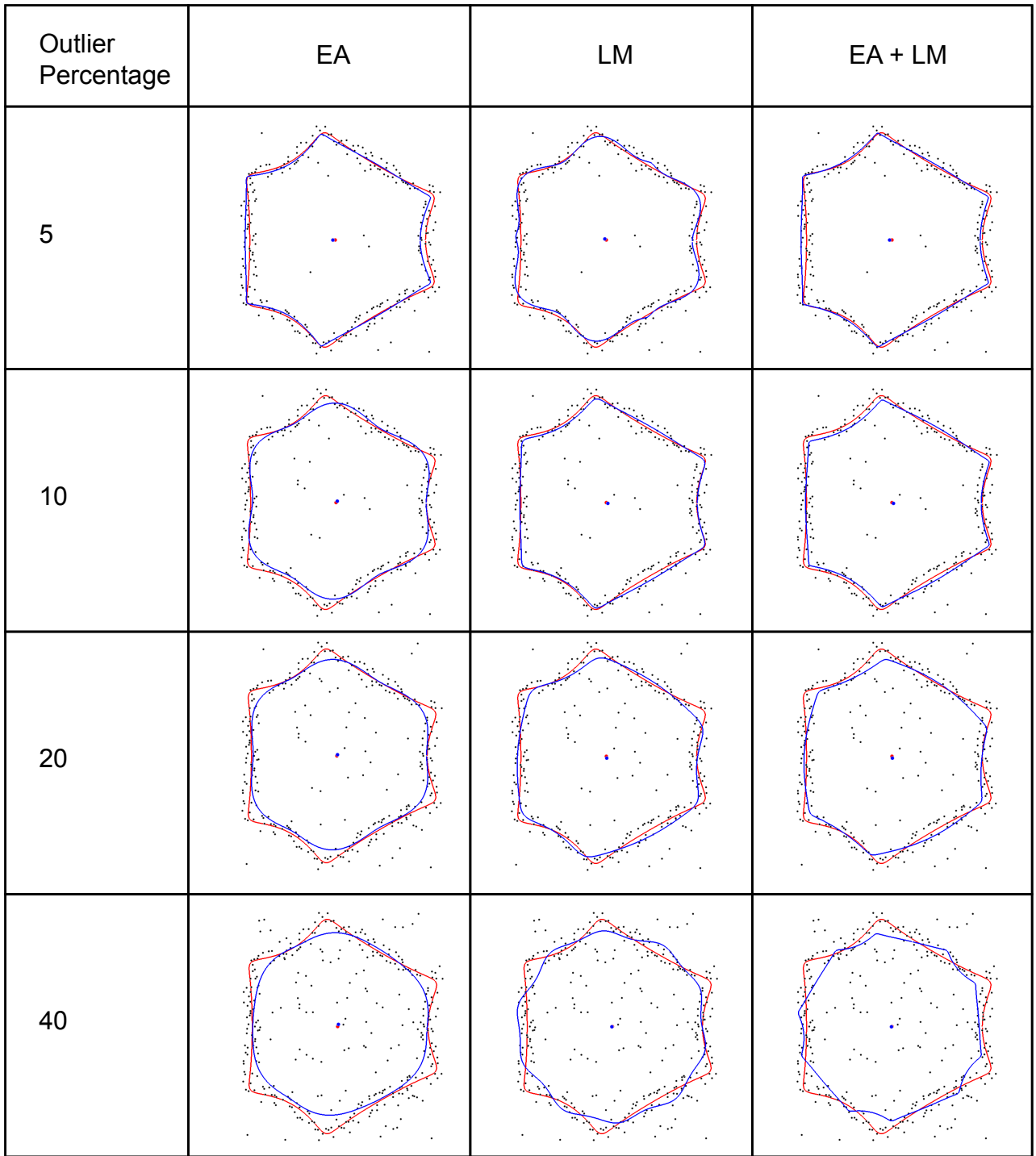

Supplement: Table S2 — Robustness to outliers. (ZIP) [file pone.0029324.s002.zip › Table S2.pdf]
