# Supplementary material for: Universal Natural Shapes: From Unifying Shape Description to Simple Methods for Shape Analysis and Boundary Value Problems
Source: PLoS One. 2012 Sep 27;7(9):e29324. doi: 10.1371/journal.pone.0029324 (PMC3459917; doi:10.1371/journal.pone.0029324)
Supplement: Table S3 — Computational times for evolutionary and deterministic algorithms, in seconds. (ZIP) [file pone.0029324.s003.zip › Table S3.docx]

| Point Number | GA using fields 7-9 | GA using SED | Levenberg-Marquardt |
| --- | --- | --- | --- |
| 250 | 0,001 | 0,0055 | 0,7 |
| 500 | 0,002 | 0,011 | 1,2 |
| 750 | 0,003 | 0,016 | 1,6 |
| 1000 | 0,004 | 0,022 | 1,9 |
| Table 3: Computational times for evolutionary and deterministic algorithms, in seconds. | | | |
